# Is there indirect selection on female extra‐pair reproduction through cross‐sex genetic correlations with male reproductive fitness?

**DOI:** 10.1002/evl3.56

**Published:** 2018-06-15

**Authors:** Jane M. Reid, Matthew E. Wolak

**Affiliations:** ^1^ School of Biological Sciences University of Aberdeen Aberdeen United Kingdom; ^2^ Department of Biological Sciences Auburn University Auburn Alabama 36849

**Keywords:** Additive genetic variance, heritability, lifetime reproductive success, mating system evolution, polyandry, quantitative genetics, sexual conflict

## Abstract

One key hypothesis explaining the evolution and persistence of polyandry, and resulting female extra‐pair reproduction in socially monogamous systems, is that female propensity for extra‐pair reproduction is positively genetically correlated with male reproductive fitness and consequently experiences positive cross‐sex indirect selection. However, key genetic correlations have rarely been estimated, especially in free‐living populations experiencing natural (co)variation in reproductive strategies and fitness. We used long‐term life‐history and pedigree data from song sparrows (*Melospiza melodia*) to estimate the cross‐sex genetic correlation between female propensity for extra‐pair reproduction and adult male lifetime reproductive success, and thereby test a key hypothesis regarding mating system evolution. There was substantial additive genetic variance in both traits, providing substantial potential for indirect selection on female reproductive strategy. However, the cross‐sex genetic correlation was estimated to be close to zero. Such small correlations might arise because male reproductive success achieved through extra‐pair paternity was strongly positively genetically correlated with success achieved through within‐pair paternity, implying that the same successful males commonly sire offspring produced by polyandrous and monogamous females. Cross‐sex indirect selection may consequently have limited capacity to drive evolution of female extra‐pair reproduction, or hence underlying polyandry, in systems where multiple routes to paternity success exist.

**Impact summary**

Why do females commonly mate with multiple males when a single mating would seemingly suffice to fertilize a female's eggs and hence ensure her reproductive success? Such female multiple mating, known as polyandry, is widely observed across the animal kingdom but is hard to explain because multiple mating is often harmful for females.

One interesting idea is that females are caught in an evolutionary bind resulting from their reproductive interactions with males. Specifically, because males are likely to increase their total reproductive success by mating with multiple females, genes that cause males to mate multiply are likely to be favored by selection. Further, males that mate extensively are likely to produce offspring with females that are also willing to mate multiply (i.e., that are polyandrous). These offspring will inherit genes for multiple mating from their mother and genes for high reproductive success from their father, causing these sets of genes to become associated. Selection that causes genes for high male reproductive success to spread through a population might consequently cause genes for polyandry to spread too.

However, the key idea that genes underlying female multiple mating are associated with genes underlying high male reproductive success has not yet been tested in wild populations where individuals are free to mate as they choose. We analyzed long‐term data from song sparrows, where females and males form socially monogamous breeding pairs but both sexes commonly also mate with other individuals. We discovered that female extra‐pair reproduction and male reproductive success both have a substantial genetic basis. However, there was no association between genes that increase female extra‐pair reproduction and genes that increase male reproductive success. Consequently, our study does not support the idea that female promiscuity is a side‐product of selection on males.

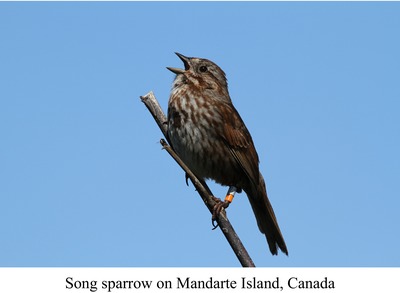


One general hypothesis explaining the evolution and persistence of complex reproductive strategies, and of resulting mating systems, is that key reproductive traits expressed in one sex experience indirect selection stemming from genetic correlations with traits expressed and directly selected in the other sex (Burt 1995; Kirkpatrick and Barton 1997; Cordero and Eberhard 2003; House et al. 2008; Forstmeier et al. 2011; Neff and Svensson 2013; Gosden et al. 2014; Moorad and Walling 2017). Such cross‐sex indirect selection is particularly pertinent when traits expressed by the focal sex are known or suspected to experience negative direct selection, and consequently defy straightforward evolutionary explanation.

One well‐known example concerns polyandry, defined as female mating with multiple males within a single reproductive episode, commonly resulting in multiple or extra‐pair paternity within single batches of offspring. Polyandry can experience positive direct selection, for example when multiple mating ensures female fertility and/or additional males provide cumulative resources that increase female fecundity (Jennions and Petrie 2000; Simmons 2005; Slatyer et al. 2012; Egan et al. 2016). However, such effects often appear to be weak or absent, and numerous sources of negative direct selection against polyandry have been demonstrated or hypothesized (e.g., stemming from physical harm, time or energy expenditure and/or predation or disease risk to females, Keller and Reeve 1995; Jennions and Petrie 2000; Cordero and Eberhard 2003; Simmons 2003, 2005; Evans and Simmons 2008; Slatyer et al. 2012; Parker and Birkhead 2013). Further, paternity loss might cause an additional component of negative selection by prompting reduced male care for polyandrous females’ offspring (Arnqvist and Kirkpatrick 2005; Kokko and Jennions 2008; Neff and Svensson 2013). Explaining the evolution and persistence of polyandry, and resulting extra‐pair paternity, consequently remains a core problem in evolutionary ecology (Slatyer et al. 2012; Pizzari and Wedell 2013; Parker and Birkhead 2013; Forstmeier et al. 2014). One pertinent hypothesis is that female propensity for multiple mating, and/or for resulting extra‐pair reproduction, is positively genetically correlated with components of male fitness and hence experiences positive cross‐sex indirect selection (Halliday and Arnold 1987; Keller and Reeve 1995; Arnqvist and Kirkpatrick 2005; Evans and Simmons 2008; Forstmeier et al. 2011, 2014; Neff and Svensson 2013; Reid et al. 2014a; Egan et al. 2016; Travers et al. 2016).

Such positive genetic correlations could result from pleiotropic effects of alleles at specific loci that influence both female and male propensities for multiple mating, potentially generating intralocus sexual conflict (Halliday and Arnold 1987; Forstmeier et al. 2011, 2014; Neff and Svensson 2013; Zietsch et al. 2015). Further, because polyandry commonly affects distributions of paternity, genetic correlations could result from linkage disequilibria that arise among alleles at physically unlinked loci. For example, there might be inevitable assortative mating, and hence assortative reproduction, between polyandrous females and promiscuous males, causing alleles underlying these sex‐specific reproductive behaviors to become associated in resulting offspring (e.g., Arnqvist and Kirkpatrick 2005; Reid et al. 2014a). Similarly, alleles underlying polyandry might become associated with alleles that increase male fertilization success given the sperm competition caused by polyandry (i.e., the “sexually selected sperm” hypothesis), and potentially with alleles that increase other components of male fitness (i.e., the general “good sperm” hypothesis, Keller and Reeve 1995; Yasui 1997; Simmons 2003, 2005; Evans and Simmons 2008; Iyengar and Reeve 2010; Egan et al. 2016; Travers et al. 2016). Such linkage disequilibria may typically be weak, and evolutionary responses may be further constrained by genetic architectures (e.g., sex‐linkage) that limit father–son inheritance of key reproductive traits (Kirkpatrick and Barton 1997; Pizzari and Birkhead 2002; Simmons 2003; Kirkpatrick and Hall 2004; Arnqvist and Kirkpatrick 2005; Bocedi and Reid 2015). Yet, in some circumstances, such disequilibria might cause sufficient indirect selection to counteract weak negative direct selection against polyandry, or reinforce positive direct selection, thereby facilitating polyandry evolution (Kirkpatrick and Hall 2004; Kokko et al. 2006; Iyengar and Reeve 2010; Egan et al. 2016).

However, such hypotheses explaining evolution of female multiple mating (and resulting multiple or extra‐pair paternity) through intrinsic cross‐sex genetic correlations with male reproductive traits rest on a critical assumption that relevant male traits, such as mating rate or fertilization success, are positively genetically correlated with male fitness and hence experience strong positive direct selection (Halliday and Arnold 1987; Kirkpatrick and Barton 1997; Arnqvist and Kirkpatrick 2005; House et al. 2008). This condition is plausible, but is not inevitable because such male traits may trade‐off against each other and against other components of fitness (e.g., Kokko et al. 2006; Evans and Simmons 2008; Evans 2010; Parker and Birkhead 2013). For example, in socially monogamous systems, male propensity for extra‐pair mating might be negatively genetically correlated with ability to defend within‐pair paternity or deliver paternal care, reflecting a trade‐off in time or energy allocation (e.g., Kokko and Jennions 2008; Lyu et al. 2017). A male's extra‐pair reproductive success accrued with polyandrous females might then be negatively genetically correlated with within‐pair reproductive success, potentially causing net negative selection against male promiscuity. Indeed, the occurrence of polyandry can reduce a male's probability of fertilization success given (i.e., conditional on) mating, and hence reduce the fitness benefit of multiple mating (i.e., the male Bateman gradient, Parker and Birkhead 2013). Further, promiscuity might potentially reduce male survival, and hence reduce future within‐pair and/or extra‐pair reproductive success. Given the potential for such trade‐offs, theoretical expectations, or observations, of positive genetic correlations between female polyandry or extra‐pair reproduction and any focal male reproductive trait do not prove that there will be net positive cross‐sex indirect selection on the female strategy, or resulting evolution (e.g., Kokko et al. 2006). Moreover, observations of positive genetic correlations between any focal male trait and total male reproductive success also do not prove that there will be consequent evolution of polyandry. This is because further genetic constraints that impede rather than drive evolution could potentially arise in multidimensional trait‐space when males can achieve reproductive success through multiple routes (e.g., Walsh and Blows 2009; Reid et al. 2014a, b; Walling et al. 2014). Ultimately, therefore, empirical test of the overarching hypothesis that evolution or persistence of female reproductive strategy is facilitated by indirect selection resulting from cross‐sex genetic correlations with male reproductive fitness requires explicit estimation of genetic correlations between key female traits and total male reproductive success.

Cross‐sex genetic correlations are notoriously difficult to estimate precisely (Lynch 1999; Bonduriansky and Chenoweth 2009), particularly for sex‐limited traits in dioecious species. Here, because female and male traits are expressed in different individuals, genetic correlations must be estimated from among‐individual phenotypic associations, substantially reducing the variance in relatedness and resulting statistical power below that available when both traits of interest can be observed in the same individual (i.e., within‐individual phenotypic associations are also observable). Key parameters are perhaps most readily estimated using large structured breeding designs, or inferred from replicated experimental evolution. However, power is often still low, generating substantial uncertainty (e.g., Forstmeier et al. 2011; Gosden et al. 2014; Punzalan et al. 2014; Calsbeek et al. 2015). Further, imposing structured breeding designs might erode linkage disequilibria and resulting genetic correlations that would arise given natural patterns of assortative reproduction, while evolutionarily relevant measures of male reproductive success are hard to obtain in such constrained environments (Cordero and Eberhard 2003; Garcia‐Gonzalez and Evans 2010; Reid 2015). Experimental studies must therefore be complemented by estimates of cross‐sex genetic correlations between key female reproductive traits and male reproductive success expressed in wild populations experiencing natural (co)variation in reproductive strategy and fitness (e.g., Lynch 1999; Brommer et al. 2007; Kruuk et al. 2008).

We used comprehensive pedigree and life‐history data from free‐living socially monogamous but genetically polygynandrous song‐sparrows (*Melospiza melodia*) to quantify the cross‐sex genetic correlation between female extra‐pair reproduction per brood (EPR), a key reproductive trait that results from polyandry, and adult male lifetime reproductive success (LRS). We thereby test the key hypothesis that female propensity for EPR experiences positive cross‐sex indirect selection through total male reproductive success. Further, we quantified the within‐sex genetic correlation between male lifetime within‐pair reproductive success (LWPRS) and lifetime extra‐pair reproductive success (LEPRS), and thereby elucidate how the cross‐sex genetic correlation between female EPR and male LRS could be shaped by the genetic structure of male reproductive success.

## Methods

### STUDY SYSTEM

Long‐term data from song sparrows resident on Mandarte Island, BC, Canada, have proved valuable for estimating additive genetic variances (V_A_), covariances (COV_A_), and correlations (r_A_) in and among sex‐specific reproductive traits and fitness components (Reid et al. 2011a, b, 2014a, b; Reid 2012; Reid and Sardell 2012, Supporting Information S1). Briefly, since 1975, almost all breeding attempts were closely monitored and all chicks surviving to ca. 6 days posthatch and adult immigrants (∼1 year^−1^ on average) were individually color‐ringed (Smith et al. 2006). The identities of the socially paired female and male that reared each brood, and of any males that remained socially unpaired due to a male‐biased adult sex ratio, were recorded (Smith et al. 2006; Lebigre et al. 2012). All chicks ringed since 1993, and all potential parents, were blood‐sampled and genotyped at ∼160 microsatellite markers. Paternities were subsequently assigned with extremely high individual‐level confidence, effectively eliminating paternity error (Sardell et al. 2010; Nietlisbach et al. 2017; Supporting Information S2). Overall, ca. 28% of chicks representing ca. 44% of broods were sired by extra‐pair males, demonstrating frequent EPR (Sardell et al. 2010). Previous analyses demonstrated nonzero V_A_ in female propensity for EPR (Reid et al. 2011a, 2014a) and in components of male paternity success (Reid et al. 2014b). However, the key hypothesis that female EPR is positively genetically correlated with total male reproductive success, and could consequently experience positive indirect selection, has not been tested in song sparrows or any other free‐living population.

### QUANTITATIVE GENETIC ANALYSES

We fitted a bivariate “animal model” (i.e., a generalized linear‐mixed model that utilizes an additive genetic relatedness matrix derived from pedigree data) to estimate V_A_ in female EPR and adult male LRS and the cross‐sex COV_A_, and thereby compute the cross‐sex r_A_ (Supporting Information S3). Since distributions of EPR and LRS are non‐Gaussian, parameters were estimated on latent scales, thereby fulfilling the fundamental quantitative genetic assumption of multivariate normality of additive genetic effects.

To quantify female EPR, the numbers of extra‐pair and total offspring were recorded for each brood where ≥1 offspring survived to paternity assignment (i.e., 6 days posthatch). Individual females produced 1–3 broods/year (mean 1.9 ± 0.6 SD, median 2) across reproductive lifespans of 1–8 years (mean 2.2 ± 1.5 SD, median 2). As with most field systems, a female's degree of polyandry (i.e., her number of mates per reproductive episode) is not readily observable. However female EPR per brood, which is observable, is a key trait in the context of mating system evolution since it is distributions of paternity, not matings *per se*, that could generate linkage disequilibria and resulting genetic correlations with components of male reproductive success (Arnqvist and Kirkpatrick 2005; Reid et al. 2011a). Adult male LRS was measured as the total number of 6‐day‐old offspring that a male sired during his adult lifetime (i.e., from reproductive maturity at age one year, see *Discussion* and Supporting Information S3).

The model structure for female EPR (measured per brood) included random year effects, and random individual female effects to account for nonindependence among broods and estimate “permanent individual” variance (reflecting “permanent environmental” and/or nonadditive genetic effects). It also included random social mate effects to capture effects of a female's socially paired male on expression of EPR in each breeding attempt (Reid et al. 2014a). Female EPR does not vary markedly with female age in our system (Reid et al. 2011a). The model structure for male LRS included random cohort (i.e., natal year) effects, but not random individual male effects because LRS is observed once per male. Residual variances were estimated for both traits, with residual covariance fixed to zero since female EPR and male LRS are not expressed by the same individual (Supporting Information S3). Fixed effects were restricted to trait‐specific regressions on individual coefficient of inbreeding (*f*), thereby accounting for resemblance among relatives resulting from correlations in *f*, and estimating trait‐specific inbreeding depression (Reid and Keller 2010).

Phenotypic data for female EPR comprised all individual broods observed during 1993–2015 (i.e., the period of genetic paternity assignment). Phenotypic data for male LRS were restricted to males hatched during 1992–2010, all of which had died by 2016. The complete genetically assigned LRS for all males from these cohorts is therefore known with probably no error or missing data. All males hatched after 2010 were excluded because some individuals from these cohorts were still alive in 2016, meaning that LRS was not fully measured. Consequently, to utilize all available data on male reproductive success, we fitted a second bivariate animal model that estimated V_A_ in female EPR (again measured per brood) and adult male annual reproductive success (ARS, i.e., the total number of offspring that a male sired in any one year), and estimated the cross‐sex COV_A_ and hence r_A_. This analysis included all observations of ARS for all adult males alive in each year during 1993–2015, irrespective of their hatch year. Since V_A_ in male LRS primarily reflects V_A_ in ARS rather than longevity (Wolak et al. 2018), ARS is an informative proxy for LRS. Model structure was as above, except the model for male ARS included random individual male effects and random year (rather than cohort) effects, and estimated the cross‐sex year covariance to capture any common year effects on EPR and ARS. The model also included fixed effects of male age category (1, 2–5 or 6+ years) to account for age‐specific variation in ARS (Keller et al. 2008).

An adult male's total LRS comprises his lifetime within‐pair reproductive success (LWPRS, i.e., total offspring sired with his socially paired females) plus his lifetime extra‐pair reproductive success (LEPRS, i.e., total offspring sired with polyandrous extra‐pair females). All else being equal, a positive cross‐sex genetic correlation between female EPR and male LEPRS might be expected, due to inevitable assortative reproduction between polyandrous females and successful extra‐pair sires (e.g., Arnqvist and Kirkpatrick 2005). However, this correlation, and hence the overall genetic correlation between female EPR and male LRS, will also depend on the genetic correlation between male LEPRS and LWPRS and thus on the additive genetic value for LEPRS of males that sire within‐pair offspring (which include offspring of females with low additive genetic value for EPR). To examine such effects, we fitted a third bivariate animal model to estimate V_A_ in male LEPRS and LWPRS and the within‐sex COV_A_ and r_A_. This model also estimated cohort and residual variances in both traits and associated covariances, and again included trait‐specific regressions on individual *f*.

### ANALYSIS IMPLEMENTATION

Standard algorithms were used to compute the inverse relatedness matrix and individual *f* values from the population pedigree pruned to phenotyped individuals and their known ancestors (Supporting Information S2). Bayesian animal models were fitted to facilitate estimation of V_A_, COV_A_, and r_A_ and associated uncertainty given non‐Gaussian trait distributions, using package MCMCglmm (Hadfield 2010) in R (v3.3.3, R Core Team 2017) with relatively uninformative priors (Supporting Information S3). Female EPR was modeled as a binomial trait with the numbers of extra‐pair and total offspring per brood as numerator and denominator, and logit link function. Male traits were modeled assuming Poisson distributions with log link functions and additive overdispersion. Posterior means and 95% highest posterior density credible intervals (95%CI) for key parameters were estimated across thinned samples of marginal posterior distributions. Further details of pedigrees, models, and priors are in Supporting Information S2–S4. Conclusions remained unchanged when analyses were repeated using relative (i.e., mean‐standardized) rather than absolute male LRS and ARS (Supporting Information S5).

## Results

**Figure 1:**
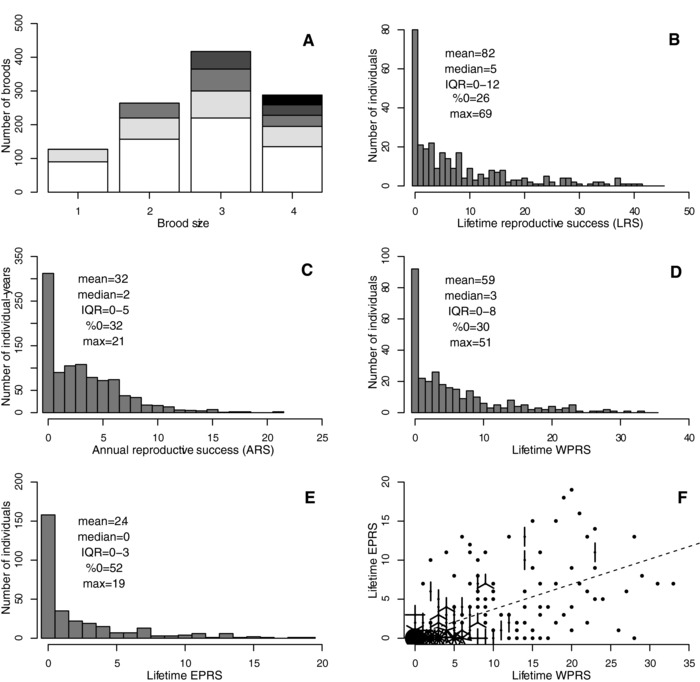
Phenotypic distributions of (A) female extra‐pair reproduction with respect to brood size, and adult male (B) lifetime reproductive success, (C) annual reproductive success, (D) lifetime within‐pair reproductive success (LWPRS) and (E) lifetime extra‐pair reproductive success (LEPRS), and (F) the phenotypic relationship between LWPRS and LEPRS across individual males. On A, white, light gray, mid gray, dark gray, and black respectively denote zero, one, two, three, and four extra‐pair offspring within a brood of each size. On F, points denote single datapoints, line segments denote further identical datapoints and the dashed line shows the linear regression of LEPRS on LWPRS (phenotypic correlation coefficient: 0.63). On B–E, the mean, median, interquartile range (IQR), percentage of observations that were zero (%0) and maximum (max) are shown. To facilitate visualization, one male with very high (i.e., max) reproductive success is not plotted on B, D, or F.

Female EPR was observed for 1096 breeding attempts made by 279 individual females, and LRS was observed for 306 adult males (Fig. 1A and B). The bivariate animal model for female EPR and male LRS estimated substantial V_A_ in both traits (Table 1A). However, the posterior mean cross‐sex COV_A_ and r_A_ were close to zero (although the 95%CIs were wide, Table 1A). There was strong inbreeding depression in male LRS, but not in female EPR (Table 1A).

**Table 1:** Bivariate models for (A) female extra‐pair reproduction (EPR) and adult male lifetime reproductive success (LRS), (B) female EPR and adult male annual reproductive success (ARS), and (C) adult male lifetime extra‐pair reproductive success (LEPRS) and lifetime within‐pair reproductive success (LWPRS)

	Model	V_A_	COV_A_ & r_A_	V_PI_	V_Soc_	V_Y_ or V_C_	COV_Y_ or COV_C_	V_R_	COV_R_	h^2^_lat_	β*_f_*
A)	Female EPR	1.51	COV_A_ = −0.01	0.37	0.39	V_Y_ = 0.18		3.72		0.24	−0.8
		(0.54–2.64)	(−0.48–0.53)	(0.001–1.21)	(0.001–1.02)	(0.06–0.36)	−	(2.61–4.85)	−	(0.10–0.39)	(−6.6–5.8)
	Male LRS	0.81	r_A_ = −0.01	−	−	V_C_ =0.34		1.47		0.31	−6.0
		(0.17–1.50)	(−0.47–0.49)			(0.09–0.69)		(0.87–2.05)		(0.10–0.56)	(−11.1−1.2)
B)	Female EPR	1.53	COV_A_ = 0.04	0.36	0.39	V_Y_ =0.20	COV_Y_ = −0.04	3.72		0.24	−0.8
		(0.46–2.69)	(−0.22–0.30)	(0.001–1.16)	(0.001–1.00)	(0.06–0.41)	(−0.25–0.18)	(2.66–4.88)	−	(0.09–0.40)	(−6.4–5.7)
	Male ARS	0.19	r_A_ = 0.07	0.07	−	V_Y_ = 0.38		0.45		0.18	−4.3
		(0.08–0.32)	(−0.36–0.49)	(0.001–0.17)		(0.16–0.68)		(0.34–0.56)		(0.08–0.31)	(−6.3−2.5)
C)	Male LEPRS	1.24	COV_A_ = 0.80	−	−	V_C_ = 0.37	COV_C_ = 0.20	2.38	1.51	0.31	−12.0
		(0.18–2.39)	(0.14–1.50)			(0.08–0.79)	(−0.05–0.47)	(1.20–3.55)	(0.83–2.25)	(0.09–0.59)	(−19.0–5.5)
	Male LWPRS	0.77	r_A_ = 0.79	−	−	V_C_ = 0.37		1.40		0.30	−4.3
		(0.16–1.46)	(0.57–0.95)			(0.10–0.73)		(0.77–1.93)		(0.10‐0.55)	(−8.7–0.8)

Estimates are the posterior means (and 95% credible intervals) for additive genetic variance (V_A_), covariance (COV_A_) and correlation (r_A_), permanent individual variance (V_PI_), social mate variance (V_Soc_), year variance (V_Y_) and covariance (COV_Y_), cohort variance (V_C_) and covariance (COV_C_), residual variance (V_R_) and covariance (COV_R_), latent‐scale heritability (h^2^_lat_) and slope of the regressions on individual coefficient of inbreeding (β*_f_*). Intercepts, and male age effects on ARS, are in Table S3.

ARS was observed for 987 male‐years, involving 401 individual males (Fig. 1C). The bivariate animal model for female EPR and male ARS yielded similar conclusions as for female EPR and male LRS. Specifically, there was nonzero V_A_ in both traits (Table 1B). The posterior mean cross‐sex COV_A_ and r_A_ were again close to zero, although the 95%CIs were again wide (Table 1B).

Across the 306 adult males whose LRS was observed, mean LWPRS exceeded mean LEPRS (Fig. 1D and E); on average, most male reproductive success was accrued through within‐pair paternity (reflecting the overall within‐pair and extra‐pair paternity rates of 72% and 28%). The bivariate animal model for male LEPRS and LWPRS estimated moderate V_A_ in both traits (Table 1C). The posterior mean COV_A_ and r_A_ were positive and substantial, with 95%CI limits that did not span zero or converge to one (Table 1C). Because the cohort and residual covariances were also positive (Table 1C), there was a strong positive phenotypic correlation between LEPRS and LWPRS (Fig. 1F). There was strong inbreeding depression in both traits, particularly in LEPRS (Table 1C).

## Discussion

One hypothesis explaining the evolution and persistence of female extra‐pair reproduction, and underlying polyandry, is that key female traits are intrinsically positively genetically correlated with male reproductive fitness and hence experience positive cross‐sex indirect selection (Halliday and Arnold 1987; Keller and Reeve 1995; Arnqvist and Kirkpatrick 2005; Forstmeier et al. 2011). Our analyses demonstrated substantial V_A_ in female EPR and adult male LRS in song sparrows, providing substantial potential for nonzero cross‐sex genetic correlations and resulting indirect selection (e.g., Jennions and Petrie 2000; Evans and Simmons 2008). However, the posterior mean cross‐sex COV_A_ and r_A_ were close to zero. There is consequently no clear evidence that strong cross‐sex indirect selection stemming from covariances with adult male reproductive fitness could facilitate ongoing evolution or persistence of female extra‐pair reproduction.

However, since COV_A_ and r_A_ were unsurprisingly estimated with considerable uncertainty, the existence of weak positive or negative indirect selection cannot be excluded. Yet, follow‐up simulations suggest that the song sparrow data and model structures would yield unbiased r_A_ estimates on average, and that posterior mean values of approximately zero are most likely to reflect true values of <0.25 (Supporting Information S6). The true r_A_ between female EPR and male LRS is consequently most likely to be small, limiting the magnitude of cross‐sex indirect selection on female EPR despite substantial V_A_ in male LRS. This conclusion is further supported by the posterior mean r_A_ between female EPR and male ARS, which was also close to zero, albeit also estimated with considerable uncertainty.

No previous studies have directly estimated r_A_ between female EPR, or polyandry or other associated components of female reproductive strategy, and total male reproductive success expressed under natural environmental conditions and mating regimes. However, r_A_ between the occurrence of female and male extra‐pair mating was estimated to be close to zero in humans (*Homo sapiens*) despite substantial heritability in both sexes (Zietsch et al. 2015). In experimental systems, Travers et al. (2016) estimated very low V_A_ in male offensive sperm competitiveness in *Drosophila melanogaster*, and consequently zero genetic correlation with female lifetime mating frequency. Meanwhile, selection on female propensity to remate caused detectable evolution of female mating rate in adzuki bean beetles (*Callosobruchus chinensis*), but no correlated evolution of male mating rate (Harano and Miyatake 2007). Similarly, selection on male mating frequency in stalk‐eyed flies (*Cyrtodiopsis dalmanni*) caused no correlated evolution of female mating frequency (Grant et al. 2005), and inheritance patterns observed in rattlebox moths (*Utethesia ornatrix*) imply that different genes affect female and male mating rates (Iyengar and Reeve 2010). Together, these studies and the song sparrow data imply that key cross‐sex genetic correlations in promiscuous mating systems may often be very small. However, in contrast, Forstmeier et al. (2011) estimated strong positive r_A_ between multiple measures of female and male extra‐pair behavior and paternity in zebra finches (*Taeniopygia guttata*, values commonly >0.5, although also estimated with considerable uncertainty). Such values would have been detectable with the song sparrow dataset (Supporting Information S6), and might reflect the zebra finch genome structure, where inheritance of few large linkage groups generates very strong linkage disequilibria (Forstmeier et al. 2012). Further studies on diverse taxa, that endeavor to overcome low power and potential experimental and observational artefacts (e.g., Grant et al. 2005; Simmons 2005; Harano and Miyatake 2007; Garcia‐Gonzalez and Evans 2010), are clearly required to allow stronger meta‐analytic inference of any general patterns.

Meanwhile, it is insightful to consider why r_A_ between female EPR and male LRS is apparently small in song sparrows, given that adult male LEPRS, which is a substantial component of adult male LRS, is by definition achieved through extra‐pair reproduction with polyandrous females. Some degree of assortative reproduction between females and males with high additive genetic values for EPR and LEPRS, and hence emerging genetic covariance, might therefore be expected. Since male LEPRS is strongly positively genetically correlated with male LWPRS (Table 1C), LEPRS is inevitably strongly positively genetically correlated with total male LRS (Supporting Information S7), potentially generating indirect selection on EPR. However, opposite to this logic, the strong positive r_A_ between male LEPRS and LWPRS might in fact eliminate any possible positive r_A_ between female EPR and male LRS. This is because, contrary to the proposition that extra‐pair paternity trades‐off against within‐pair paternity, successful extra‐pair song sparrow sires are also successful within‐pair sires and hence commonly sire offspring of females with low phenotypic and additive genetic values for EPR. For there to be cross‐sex indirect selection on female EPR, mean additive genetic values for fitness must differ between males that sire extra‐pair versus within‐pair offspring (Arnqvist and Kirkpatrick 2005). This cannot be substantively the case if the same males frequently sire both types of offspring. The genetic covariance structure of male reproductive fitness, as achieved through the dual within‐pair and extra‐pair routes, may therefore constrain the degree to which positive r_A_ between female EPR and male LRS could arise, and hence constrain rather than generate cross‐sex indirect selection on female EPR.

Proximately, the positive r_A_ between male LEPRS and LWPRS partly reflects a positive r_A_ between male within‐pair paternity success per brood and annual extra‐pair reproductive success (Reid et al. 2014b), and may also reflect a genetic basis to the observed phenotypic association between a male's probability of social pairing and siring extra‐pair offspring (Sardell et al. 2010). However, total male fitness comprises survival to maturity as well as subsequent adult LRS. Current analyses focused specifically on adult LRS because male reproductive success is the trait with which intrinsic positive genetic correlations with female EPR could arise through assortative reproduction (e.g., Keller and Reeve 1995; Arnqvist and Kirkpatrick 2005). In contrast, genetic correlations with survival to maturity (i.e., “viability,” and associated “good sperm” hypotheses) require further pleiotropy or linkage (e.g., Yasui 1997; Simmons 2005). Moreover, previous analyses showed that male and female juvenile survival are strongly positively genetically correlated in song sparrows, but may be weakly negatively genetically correlated with female EPR (Reid 2012; Reid and Sardell 2012) and adult male LRS (Wolak et al. 2018). Such negative associations between survival to maturity and aspects of adult reproductive success also occur in other systems (e.g., Chippindale et al. 2001; Mojica and Kelly 2010), and might help maintain V_A_ in adult male LRS that in turn shapes the form of indirect selection on female reproductive traits. Future analyses could also reveal how such genetic correlations among components of male fitness, and among components of female reproductive strategy and male fitness, might vary with environmental conditions.

Overall, our analyses do not support the hypothesized positive r_A_ between female EPR and male LRS, implying that female reproductive strategy is not currently substantively driven by positive cross‐sex indirect selection. New theory is now required to examine how the genetic covariance structure of male reproductive fitness arising in systems with multiple routes to paternity success might constrain emerging cross‐sex r_A_s, and hence constrain rather than facilitate ongoing evolution of EPR and underlying polyandry.

Associate Editor: A. Charmantier

## Supporting information

Supporting information

**S1**. Study system properties.
**S2**. Pedigree data.
**S3**. Additional model specifications and considerations.
**S4**. Additional fixed effects estimates.
**S5**. Relative male fitness.
**S6**. Bias and precision in estimates of r_A_.
**S7**. Additional models of male reproductive success.
