# Supplementary figures and images for: Is there indirect selection on female extra‐pair reproduction through cross‐sex genetic correlations with male reproductive fitness?
Source: Evol Lett. 2018 Jun 15;2(3):159–68. doi: 10.1002/evl3.56 (PMC6121835; doi:10.1002/evl3.56)

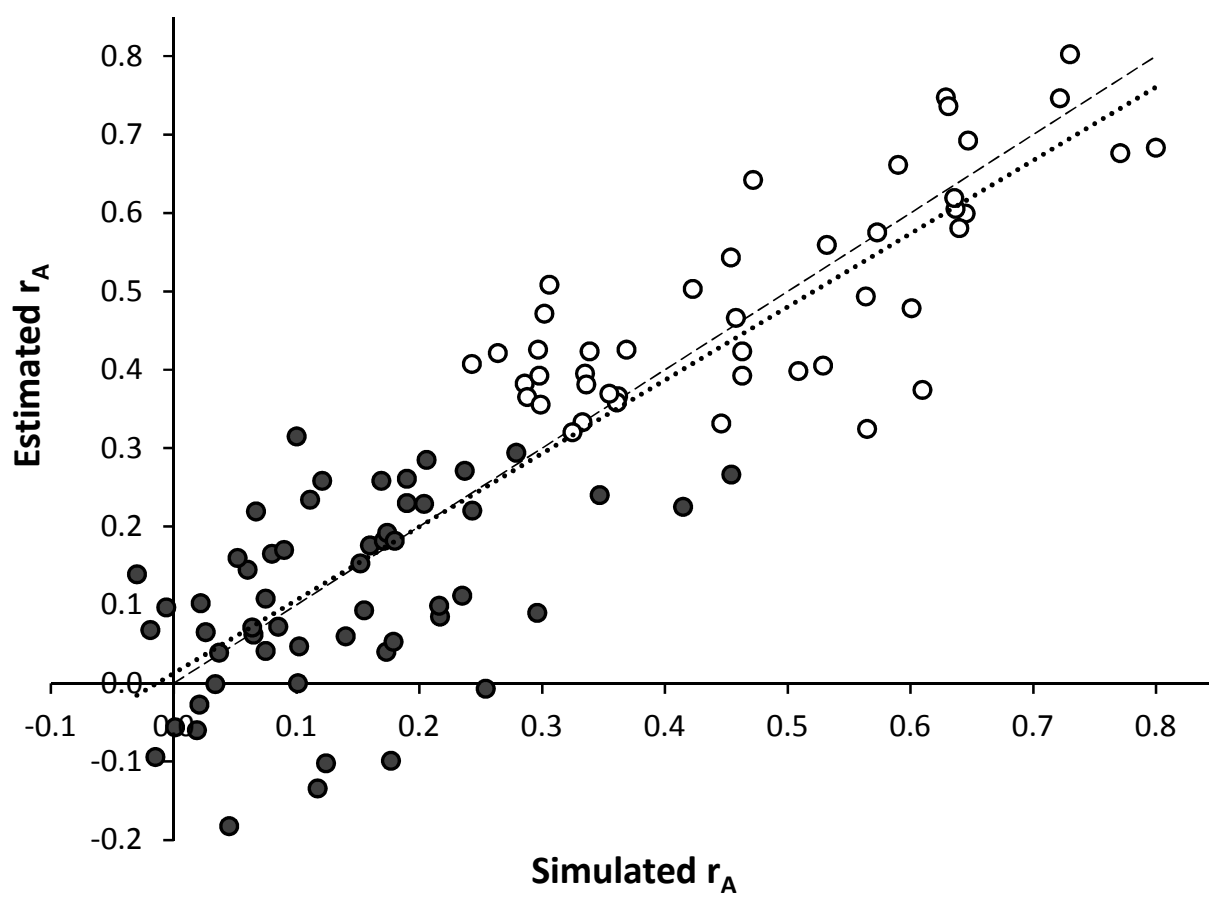

Supplement: Supplementary file 1 — Supporting information [file EVL3-2-159-s001.pdf]
