# Supplementary material for: Is there indirect selection on female extra‐pair reproduction through cross‐sex genetic correlations with male reproductive fitness?
Source: Evol Lett. 2018 Jun 15;2(3):159–68. doi: 10.1002/evl3.56 (PMC6121835; doi:10.1002/evl3.56)
Supplement: Supplementary file 2 — S1. Study system properties. S2. Pedigree data. S3. Additional model specifications and considerations. S4. Additional fixed effects estimates. S5. Relative male fitness. S6. Bias and precision in estimates of rA. S7. Additional models of male reproductive success. [file EVL3-2-159-s002.docx]

**Supporting information**

**Is there indirect selection on female extra-pair reproduction through cross-sex genetic correlations with male reproductive fitness?**

**Jane M. Reid & Matthew E. Wolak**

**S1. Study system properties**

The song sparrow study population’s small size (numbers of adults per year during 1993-2015: females: mean 28.7±11.4SD, median 29, range 11-52; males: mean 44.0±19.3SD, median 36, range 13-82) limits the number of phenotyped individuals despite the study duration. However, the system has multiple properties that facilitate estimation of additive genetic variances and covariances in sex-specific fitness-related traits, and facilitate separation of additive genetic and environmental variances.

First, there is relatively high local recruitment of both sexes (95.8% of adult females and 96.8% of adult males observed since 1993 had hatched on Mandarte), meaning that the pedigree is highly interconnected across the sexes (Reid et al. 2014a, Supporting Information S2). Second, the small population size coupled with occasional immigration generates considerable variance in relatedness (Wolak & Reid 2016, Table S2). Third, the song sparrow’s multi-brooded life-history, and frequent social repairing alongside extra-pair reproduction, generates numerous full-sibs and maternal and paternal half-sibs and other known full- and half-relatives reared in different micro-environments (Germain et al. 2018). Additive genetic effects are consequently expressed across diverse genetic and environmental backgrounds. Fourth, panmictic dispersal within Mandarte means that relatives are not spatially segregated, with no detectable spatial relatedness structure (Germain et al. 2016). Meanwhile, the occurrence of multiple overlapping generations means that close and more distant relatives overlap in time. Fifth, since all adults can be observed and adult emigration (i.e. breeding dispersal) is probably very rare, adult male lifetime reproductive success (LRS), annual reproductive success (ARS), lifetime extra-pair reproductive success (LEPRS) and lifetime within-pair reproductive success (LWPRS) can be comprehensively measured with little or no error or missing data, including males with zero reproductive success (Lebigre et al. 2012).

**S2. Pedigree data**

Pedigree data were compiled by initially assigning song sparrow chicks hatched since 1975 to their observed social parents. All available genetic paternity data were then used to correct the pedigree for known extra-pair paternity, providing complete correction during 1993-2015 and partial correction during 1989-1992 (Reid et al. 2014c; Nietlisbach et al. 2015, 2017). Since genetic parentage analyses for 1993-2008 revealed zero extra-pair maternity (Sardell et al. 2010), all pedigree maternities back to 1975 are probably correct. Some pre-1993 paternity error presumably remains. However, utilising all available pedigree information provides a more powerful analysis than the alternative assumption that all 1992 parents were unrelated (Reid et al. 2011b).

Tables S1 and S2 summarise the observed distributions of individual coefficients of inbreeding (*f*), and pairwise coefficients of kinship (*k*), calculated from the pedigree. Because immigrants’ *f* values are undefined relative to the pedigree baseline, phenotypic observations of immigrants were excluded from analyses.

**Table S1.** Statistics describing the distributions of individual coefficients of inbreeding (*f*) across focal phenotyped individuals for (A) female extra-pair reproduction (EPR), (B) male lifetime reproductive success (LRS) and (C) male annual reproductive success (ARS). N is the number of phenotyped individuals. SD and IQR are the standard deviation and inter-quartile range respectively.

|  | N | Mean ± SD | Median | IQR | Range |
| --- | --- | --- | --- | --- | --- |
| A) Female EPR | 279 | 0.067 ± 0.047 | 0.064 | 0.033-0.093 | 0.000-0.277 |
| B) Male LRS | 306 | 0.064 ± 0.043 | 0.059 | 0.037-0.082 | 0.000-0.265 |
| C) Male ARS | 401 | 0.066 ± 0.052 | 0.059 | 0.034-0.086 | 0.000-0.309 |

**Table S2.** Total numbers of phenotyped individuals (N_phen_) and individuals retained in the pruned pedigree (N_ped_), and statistics describing the distributions of pairwise coefficients of kinship (*k*) between all phenotyped individuals, for the bivariate analyses of (A) female extra-pair reproduction (EPR) and male lifetime reproductive success (LRS), (B) female EPR and male annual reproductive success (ARS), and (C) male lifetime extra-pair reproductive success (LEPRS) and lifetime within-pair reproductive success (LWPRS). SD and IQR are the standard deviation and inter-quartile range respectively.

|  | N_phen_ | N_ped_ | Mean ± SD | Median | IQR | Range |
| --- | --- | --- | --- | --- | --- | --- |
| A) EPR & LRS | 585 | 762 | 0.073 ± 0.037 | 0.067 | 0.051-0.085 | 0.005-0.401 |
| B) EPR & ARS | 680 | 822 | 0.072 ± 0.038 | 0.066 | 0.050-0.085 | 0.000-0.440 |
| C) LEPRS & LWPRS | 306 | 624 | 0.073 ± 0.036 | 0.067 | 0.053-0.085 | 0.006-0.378 |

**S3. Additional model specifications and considerations**

Animal models allow key quantitative genetic parameters, and associated uncertainty, to be estimated given natural relatedness structures and patterns of non-random mating and selection arising in free-living populations (Kruuk 2004). Models were run with 6005000 iterations, burn-in 5000 and thinning interval 3000 to achieve low (<0.1) autocorrelation among 2000 retained samples. Priors on fixed effects were normally distributed and diffuse (mean 0, variance 10^8^). Separate models were run with three different priors on variance components and variance-covariance matrices, and posterior distributions were quantitatively similar given these different specifications. Key MCMCglmm parameter values for the three sets of priors are below. In summary, prior 1 comprised inverse gamma distributions on univariate variance components and inverse Wishart distributions on bivariate variance components. Prior 2 included parameter expansion on univariate and bivariate variance components, extending the basic formulation of non-central scaled F-distributions with numerator and denominator degrees of freedom of one. Prior 3 included parameter expansion and gave approximately uniform marginal prior distributions on correlations, and hence was relatively uninformative on the genetic correlation (r_A_). Priors on residual (co)variance matrices were inverse Wishart distributed, with limit variance V=1 and degree of belief nu=1.002 in all cases.

Prior 1:

Variance-covariance matrices (bivariate): V=diag(2), nu=1.002

Variance components (univariate): V=1, nu=0.002

Prior 2:

Variance-covariance matrices (bivariate): V=diag(2), nu=1.002, alpha.mu = c(0, 0), alpha.V = diag(2)*1000

Variance components (univariate): V = 1, nu = 1, alpha.mu = 0, alpha.V = 1000

Prior 3:

Variance-covariance matrices (bivariate): V = diag(2)*0.02, nu = 3, alpha.mu = c(0, 0), alpha.V = diag(2)*1000

Variance components (univariate): V = 1, nu = 1, alpha.mu = 0, alpha.V = 1000

Posterior distributions of genetic correlations (r_A_) were computed by applying the standard formula (i.e. r_A_ = COV_A1.2_/√(V_A1_ x V_A2_), where COV_A1.2_ is the additive genetic covariance of traits 1 and 2 and V_A1_ and V_A2_ are the traits’ additive genetic variances) to each retained posterior sample for V_A1_, V_A2_ and COV_A1.2_. Although the current primary aim was not to consider heritabilities, for reference, the latent-scale heritability (h^2^_lat_) of each trait was computed as the ratio of V_A_ to the sum of all estimated variance components across each retained posterior sample. Posterior means and 95%CIs for r_A_ and h^2^_lat_ were then calculated from their respective posterior distributions.

*Trait definitions and dependencies*

Female extra-pair reproduction (EPR) was measured per brood, not per year or per female lifetime. The per-brood definition is appropriate given the definition of polyandry as the number of mates per reproductive episode (not per year or lifetime), and allows brood-specific social mate effects to be properly accounted for (e.g. Reid et al. 2014a). Male lifetime reproductive success was defined as the number of hatched offspring produced by each male, not the number of recruited offspring, since such cross-generation measures of fitness cannot be easily interpreted in quantitative genetic analyses. Genetic covariances between female EPR and juvenile survival have been considered previously (see *Discussion*, and Reid 2012; Reid & Sardell 2012).

Occurrences of extra-pair and within-pair paternity are not independent across males because extra-pair paternity by one male requires loss of within-pair paternity by another male (Reid et al. 2014b). Similarly, occurrences of male within-pair and extra-pair paternity are not independent of the degree of female extra-pair reproduction. Multinomial quantitative genetic models specified at the level of the paternity (i.e. sire identity) of each individual offspring could potentially fully account for all such phenotypic dependencies, but it is not currently feasible to fit such high-dimension models. Models were consequently fitted at the level of observed female and male traits. However, dependencies between individual males’ realised values of LEPRS and LWPRS, and between these traits and male LRS and female EPR, are likely to be small. This is because LEPRS, LWPRS and LRS are realised across multiple breeding attempts, and often across multiple years. They therefore depend on male lifespan and total female fecundity in relevant years (which in turn depends on female population size and environmental conditions), as well as on paternity realised by numerous other contemporary males. Residual covariances between female EPR and male traits were consequently assumed to be zero. Previous analyses also suggested that estimated V_A_ in female EPR does not reflect pre-observation mortality of offspring sired by different males (Reid et al. 2011a).

*Parental environmental variances*

In general, estimates of V_A_ can be inflated by parental environmental effects that increase phenotypic resemblance among siblings (Kruuk & Hadfield 2007). Power to distinguish permanent individual, maternal environmental and social paternal environmental variances for adult traits is relatively low in the song sparrow dataset, because many parents only contribute one adult son or daughter to the phenotypic dataset (Reid et al. 2014a). Specifically, the 279 adult females whose reproduction was observed were reared by totals of 155 mothers and 142 social fathers, of which 86 (55%) and 72 (53%) respectively contributed only one daughter. Similarly, the 306 adult males whose LRS was observed were reared by totals of 156 mothers and 156 social fathers, of which 81 (52%) and 84 (54%) respectively contributed only one son. Permanent individual variances and parental environmental variances are consequently partly non-identifiable. However, this structure also means that estimates of V_A_ are unlikely to be substantively inflated by parental effects. Indeed, key estimates of V_A_, COV_A_ and r_A_ in and among female EPR and measures of male reproductive success remained similar when maternal and paternal environmental variances were additionally modelled (see also Reid et al. 2014a).

*Sex linkage*

In principle, sexual conflict resulting from cross-sex genetic correlations can be resolved by evolution of sex-linkage (Bonduriansky & Chenoweth 2009), thereby negating mechanisms that are hypothesised to generate indirect selection on female reproductive traits (e.g. Pizzari & Birkhead 2002; Evans & Simmons 2008). Power to estimate autosomal versus sex-linked genetic effects for sex-limited traits is inevitably low in the song sparrow dataset, because elements of the autosomal and sex-chromosomal inverse relationship matrices are highly correlated (correlation coefficient: 0.89). However, both male LRS and female EPR are likely to be highly polygenic, and hence unlikely to be fully sex-linked. Indeed, song sparrows carry 38-40 chromosomes, and the sex-chromosomes account for a small proportion of total genome size (Nietlisbach et al. 2015).

**S4. Additional fixed effects estimates**

**Table S3.** Additional fixed effects estimates (posterior mean and 95% credible interval) from the bivariate animal models for (A) female extra-pair reproduction (EPR) and male lifetime reproductive success (LRS), (B) female EPR and male annual reproductive success (ARS), and (C) male lifetime extra-pair reproductive success (LEPRS) and lifetime within-pair reproductive success (LWPRS). For male ARS, effects for age classes 1 year and 2-5 years are contrasts from age class 6+ years.

|  | Model | Intercept | Male age 1 | Male age 2-5 |
| --- | --- | --- | --- | --- |
| A) | Female EPR | -1.89 (-2.82- -0.99) | _______________ | _______________ |
|  | Male LRS | 1.29 (0.50- 2.09) |  |  |
| B) | Female EPR | -1.88 (-2.74- -0.97) |  |  |
|  | Male ARS | 0.79 (0.36- 1.26) | -0.56 (-0.86- -0.26) | 0.53 (0.26- 0.80) |
| C) | Male LEPRS | -0.11 (-1.06- 0.83) | _______________ | _______________ |
|  | Male LWPRS | 0.86 (0.05- 1.59) |  |  |

**S5. Relative male fitness**

Quantitatively, the evolutionary response in female propensity for extra-pair reproduction (EPR, i.e. on the latent scale) stemming from cross-sex indirect selection can be directly estimated as the additive genetic covariance (COV_A_) with relative male fitness (i.e. standardised to a mean of one), rather than absolute male fitness (i.e. the ‘secondary theorem of selection’; e.g. Rausher 1992; Morrissey et al. 2010; Reid 2012). We consequently fitted two further bivariate animal models that directly estimated COV_A_ between female propensity for EPR and relative male lifetime reproductive success (LRS_W_) and relative annual reproductive success (ARS_W_).

Male ARS_W_ was calculated by dividing each observation of ARS by the mean ARS observed across all adult males alive in the relevant year. Male LRS_W_ could in principle be calculated similarly, by dividing each observation of LRS by the cohort mean. However, this form of standardisation was not appropriate for the current dataset; because few males from some cohorts survived to adulthood, dividing each observation of LRS by the cohort mean would largely standardise some males’ observations by themselves. Consequently, LRS_W_ was calculated by dividing each observation of LRS by the mean LRS across all observations. Animal model structures were the same as for the primary models presented in the main manuscript, except that Gaussian errors were assumed for male LRS_W_ and ARS_W_.

The additive genetic covariances (COV_A_), and hence the additive genetic correlations (r_A_), between female EPR and male LRS_W_ and ARS_W_ were estimated to be close to zero (Table S4), concurring with the estimates from the models of absolute male LRS and ARS (Table 1).

**Table S4.** Bivariate models for (A) female extra-pair reproduction (EPR) and male relative lifetime reproductive success (LRS_W_) and (B) female EPR and male relative annual reproductive success (ARS_W_). Estimates are the posterior means (and 95% credible intervals) for additive genetic variance (V_A_), covariance (COV_A_) and correlation (r_A_), permanent individual variance (V_PI_), social mate variance (V_Soc_), year variance (V_Y_) and covariance (COV_Y_), cohort variance (V_C_) and residual variance (V_R_), latent-scale heritability (h^2^) and slope of the regressions on individual coefficient of inbreeding (β*_f_*).

|  | Model | V_A_ | COV_A_ & r_A_ | V_PI_ | V_Soc_ | V_Y_ or V_C_ | COV_Y_ | V_R_ | h^2^ | β*_f_* |
| --- | --- | --- | --- | --- | --- | --- | --- | --- | --- | --- |
| A | Female EPR | 1.50  (0.54-2.62) | COV_A_ = 0.01  (-0.33-0.34) | 0.37  (0.001-1.13) | 0.38  (0.001-1.00) | V_Y_ = 0.18  (0.06-0.35) | ________ | 3.71  (2.55-4.80) | 0.24  (0.10-0.40) | -0.9  (-7.0- 5.4) |
|  | Male LRS_W_ | 0.31  (0.12-0.57) | r_A_ = 0.01  (-0.49-0.47) | _________ | _________ | V_C_ = 0.28  (0.09-0.56) |  | 1.16  (0.93-1.46) | 0.18  (0.08-0.33) | -3.9  (-7.3- -0.6) |
| B | Female EPR | 1.34  (0.39-2.42) | COV_A_ = 0.01  (-0.14-0.20) | 0.55  (0.001-1.28) | 0.52  (0.001-1.07) | V_Y_ = 0.19  (0.06-0.36) | -0.003  (-0.07-0.06) | 3.63  (2.55-4.66) | 0.21  (0.08-0.37) | -0.7  (-6.8- 5.6) |
|  | Male ARS_W_ | 0.11  (0.06-0.17) | r_A_ = 0.02  (-0.41-0.46) | 0.03  (<0.001-0.08) | _________ | V_Y_ = 0.08  (0.03-0.14) |  | 0.81  (0.73-0.90) | 0.11  (0.06-0.17) | -3.1  (-4.6- -1.7) |

**S6. Bias and precision in estimates of r_A_**

Interpreted directly, the 95%CI around the estimate of the cross-sex genetic correlation (r_A_) between female extra-pair reproduction (EPR) and male lifetime reproductive success (LRS, Table 1) implies that absolute r_A_ values up to ca. 0.47 cannot be excluded (Table 1). However, in multivariate animal models, precision of r_A_ estimates might increase with the true magnitude of r_A_. This is because, for genetically correlated traits, precision of V_A_ and COV_A_ (and hence r_A_) estimates can increase because phenotypic observations of each trait directly inform estimation of V_A_ for the other trait through pedigree links among all observed individuals. There is no such information transfer when r_A_ is truly zero.

Comprehensive investigations of bias and precision for multivariate animal models involving fitness traits are challenging for multiple reasons. Phenotypes must be simulated on appropriate observed scales given known genetic and environmental variances and covariances on underlying latent scales. Multivariate animal models that consider appropriate non-Gaussian distributions are consequently required to recover estimates, which currently require computationally intensive MCMC methods that impede comprehensive simulations. Further, realisations of traits such as EPR and LRS given breeding values simulated on an observed pedigree are no longer consistent with the pedigree structure (which itself reflects the original expression of EPR and LRS), and might consequently fail to capture aspects of data structure that affect analyses. Pedigree structure could potentially be simulated alongside focal phenotypes to ensure internal consistency, but the simulated pedigrees may then no longer be directly comparable with the observed pedigree unless all key rules regarding the distributions of female and male reproduction and parentage are adequately captured.

Consequently, to explore bias and precision in estimates of r_A_ given the song sparrow pedigree and data structure and animal model framework, we retained the real observed pedigree structure and distribution of phenotypic observations, and generated phenotypes from simulated breeding values and environmental deviations. Latent-scale values of V_A_ and the total environmental (residual) variance V_E_ were set to match those estimated from the real data, and COV_A_ was set to values between zero and 0.8, across 100 iterations. Breeding values were simulated using package pedantics (Morrissey & Wilson 2009) in R (v3.3.3, R Core Team 2017), and simulated phenotypic values for EPR and LRS were recovered using inverse-logit and exponential transformations respectively. The realised correlation in breeding values, which deviated slightly from that simulated due to the small population size, was extracted. Animal models were fitted as for the primary analysis of the real data, except that fewer (500) posterior samples were retained to minimise computing time. The posterior mean r_A_ estimated in each simulation, and the associated 95%CI, were extracted. Negative values of r_A_ were not simulated due to constraints on computing time. However, associated bias and precision can be inferred from positive values of the same absolute magnitude. Other variance components (i.e. year, cohort and social sire variances), and inbreeding depression, were not simulated (i.e. assumed to be zero) for simplicity. These simulations do not comprise a definitive analysis, but they serve to illustrate likely degrees of bias and precision in relation to the true magnitude of r_A_, and hence indicate true values of r_A_ that could readily generate estimated values close to zero.

The simulations showed that the song sparrow data and animal model returned unbiased estimates of r_A_ on average given the simulated data (Fig. S1). Although individual estimates were often relatively imprecise, estimates of r_A_ that were close to (or below) zero only occurred when true r_A_ was less than ca. 0.25 (Fig. S1). True values exceeding this threshold were typically detected (i.e. 95%CIs did not include zero).

**Figure S1.** Relationship between simulated values of the cross-sex genetic correlation between female extra-pair reproduction (EPR) and adult male lifetime reproductive success (LRS, simulated r_A_), and posterior mean values estimated from an animal model (estimated r_A_) across 100 simulations. Filled and open symbols respectively denote cases where the 95%CI did and did not overlap zero. The 95%CI included the simulated value in all cases. The dotted line indicates the regression of estimated r_A_ on simulated r_A_, and the dashed line indicates the expected 1:1 relationship. The regression slope did not differ from 1, or hence from expectation (0.94±0.05SE).

**S7. Additional models of male reproductive success**

Two further bivariate animal models were fitted to directly quantify the additive genetic covariance and correlation between adult male lifetime extra-pair reproductive success (LEPRS) and total lifetime reproductive success (LRS), and between lifetime within-pair reproductive success (LWPRS) and LRS. These models had the same structure as the main model for male LEPRS and LEPRS. As expected, LEPRS and LWPRS were both strongly positively genetically correlated with total LRS (Table S5).

**Table S5.** Bivariate models for adult male lifetime reproductive success (LRS) and (A) lifetime extra-pair reproductive success (LEPRS) and (B) lifetime within-pair reproductive success (LWPRS). Estimates are the posterior means (and 95% credible intervals) for additive genetic variance (V_A_), covariance (COV_A_) and correlation (r_A_), cohort variance (V_C_) and covariance (COV_C_), residual variance (V_R_) and covariance (COV_R_), and slope of the regressions on individual coefficient of inbreeding (β*_f_*). Values of r_A_ are highlighted.

|  | Model | V_A_ | COV_A_ & r_A_ | V_C_ | COV_C_ | V_R_ | COV_R_ | β*_f_* |
| --- | --- | --- | --- | --- | --- | --- | --- | --- |
| (A) | LEPRS | 1.43  (0.26-2.96) | COV_A_ = 1.00  (0.14-2.02) | 0.39  (0.10-0.86) | 0.24  (-0.04-0.58) | 2.97  (1.70-4.36) | 2.12  (1.32-3.12) | -11.9  (-18.8- -4.8) |
|  | LRS | 0.84  (0.18-1.61) | r_A_ = 0.89  (0.68-0.97) | 0.32  (0.09-0.67) |  | 1.65  (0.96-2.25) |  | -6.0  (-11.6- -1.7) |
| (B) | LWPRS | 1.06  (0.19-2.06) | COV_A_ = 1.02  (0.15-1.99) | 0.42  (0.10-0.84) | 0.31  (0.04-0.70) | 2.03  (1.23-2.97) | 1.97  (1.19-2.82) | -5.1  (-10.5- 0.6) |
|  | LRS | 1.06  (0.17-2.03) | r_A_ = 0.95  (0.82-0.98) | 0.38  (0.11-0.78) |  | 1.98  (1.24-2.85) |  | -6.7  (-12.5- -1.1) |

**References**

Bonduriansky, R. & Chenoweth, S.F. (2009). Intralocus sexual conflict. *Trends Ecol. Evol.* 24:280-288.

Evans, J.P. & Simmons, L.W. (2008). The genetic basis of traits regulating sperm competition and polyandry: can selection favour the evolution of good- and sexy-sperm? *Genetica* 134:5-19.

Germain, R., Wolak, M.E., Arcese, P., Losdat, S. & Reid, J.M. (2016). Direct and indirect genetic and fine-scale environmental effects on breeding date in song sparrows. *J. Anim. Ecol.* 85:1613-1624.

Germain, R., Arcese, P. & Reid, J.M. (2018). The consequences of polyandry for sibship structures, distributions of relationships and relatedness, and potential for inbreeding in a wild population. *Am. Nat.* online.

Kruuk, L.E.B. (2004). Estimating genetic parameters in natural populations using the “animal model.” *Phil. Trans. R. Soc. B* 359:873–890.

Kruuk, L.E.B. & Hadfield, J.D. (2007). How to separate genetic and environmental causes of similarity between relatives. *J. Evol. Biol.* 20:1890-1903.

Lebigre, C., Arcese, P., Sardell, R.J., Keller, L.F. & Reid, J.M. (2012). Extra-pair paternity and the variance in male fitness in song sparrows (*Melospiza melodia*). *Evolution* 66:3111-3129.

Morrissey, M.B. & Wilson, A.J. (2009). PEDANTICS: an R package for pedigree-based genetic simulation and pedigree manipulation, characterization and viewing. *Mol. Ecol. Res.* 10:711-719

Morrissey, M.B., Kruuk, L.E.B. & Wilson, A.J. (2010). The danger of applying the breeder’s equation in observational studies of natural populations. *J. Evol. Biol.* 23:2277-2288.

Nietlisbach, P., Camenisch, G., Bucher, T., Slate, J., Keller, L.F. & Postma, E. (2015). A microsatellite-based linkage map for song sparrows. *Mol. Ecol. Res.* 15:1486-1496.

Nietlisbach, P., Keller, L.F., Camenisch, G., Guillaume, F., Arcese, P., Reid, J.M. & Postma, E. (2017). Pedigree-based inbreeding coefficient explains more variation in fitness than heterozygosity at 160 microsatellites in a wild bird population. *Proc. R. Soc. B* 284:20162763.

Pizzari, T. & Birkhead, T.R. (2002). The sexually-selected sperm hypothesis: sex-biased inheritance and sexual antagonism. *Biol. Rev.* 77:183-209.

R Core Team. (2017). R: A language and environment for statistical computing. R Foundation for Statistical Computing, Vienna, Austria.

Rausher, M.D. (1992). The measurement of selection on quantitative traits: biases due to environmental covariances between traits and fitness. *Evolution* 46:616-626.

Reid, J.M. (2012). Predicting evolutionary responses to selection on polyandry in the wild: additive genetic covariances with female extra-pair reproduction. *Proc. R. Soc. B* 279:4652-4660.

Reid, J.M., Arcese, P., Keller, L.F. & Losdat, S. (2014a). Female and male genetic effects on offspring paternity: additive genetic (co)variances in female extra-pair reproduction and male paternity success in song sparrows (*Melospiza melodia*). *Evolution* 68:2357–2370.

Reid, J.M., Arcese, P. & Losdat, S. (2014b). Genetic covariance between components of male reproductive success: within-pair versus extra-pair paternity in song sparrows. *J. Evol. Biol.* 27:2046–2056.

Reid, J.M., Arcese, P., Sardell, R.J. & Keller, L.F. (2011b). Additive genetic variance, heritability and inbreeding depression in male extra-pair reproductive success. *Am. Nat.* 177:177-187.

Reid, J.M., Keller, L.F., Marr, A.B., Nietlisbach, P., Sardell, R.J. & Arcese, P. (2014c). Pedigree error due to extra-pair reproduction substantially biases estimates of inbreeding depression. *Evolution* 68:802-815.

Reid, J.M. & Sardell, R.J. (2012). Indirect selection on female extra-pair reproduction? Comparing the additive genetic value of maternal half-sib extra-pair and within-pair offspring. *Proc. R. Soc. B* 279:1700–1708.

Sardell, R.J., Keller, L.F., Arcese, P., Bucher, T. & Reid, J.M. (2010). Comprehensive paternity assignment: genotype, spatial location and social status in song sparrows *Melospiza melodia*. *Mol. Ecol.* 19:4352-4364.

Wolak, M.E. & Reid, J.M. (2016). Is pairing with a relative heritable? Estimating female and male genetic contributions to the degree of biparental inbreeding in song sparrows (*Melospiza melodia*). *Am. Nat.* 187:736–752.
